# Axonal neurotransmitter release in the regulation of myelination

**DOI:** 10.1042/BSR20231616

**Published:** 2024-09-25

**Authors:** Katy L.H. Marshall-Phelps, Rafael G. Almeida

**Affiliations:** 1 Centre for Discovery Brain Sciences, University of Edinburgh, Edinburgh, U.K.; 2 MS Society Edinburgh Centre for MS Research, University of Edinburgh, Edinburgh, U.K.

**Keywords:** axon, myelin, neural circuits, neuroplasticity, neurotransmission, oligodendrocytes

## Abstract

Myelination of axons is a key determinant of fast action potential propagation, axonal health and circuit function. Previously considered a static structure, it is now clear that myelin is dynamically regulated in response to neuronal activity in the central nervous system (CNS). However, how activity-dependent signals are conveyed to oligodendrocytes remains unclear. Here, we review the potential mechanisms by which neurons could communicate changing activity levels to myelin, with a focus on the accumulating body of evidence to support activity-dependent vesicular signalling directly onto myelin sheaths. We discuss recent *in vivo* findings of activity-dependent fusion of neurotransmitter vesicles from non-synaptic axonal sites, and how modulation of this vesicular fusion regulates the stability and growth of myelin sheaths. We also consider the potential mechanisms by which myelin could sense and respond to axon-derived signals to initiate remodelling, and the relevance of these adaptations for circuit function. We propose that axonal vesicular signalling represents an important and underappreciated mode of communication by which neurons can transmit activity-regulated signals to myelinating oligodendrocytes and, potentially, more broadly to other cell types in the CNS.

## Introduction

Myelin, made by oligodendrocytes in the central nervous system (CNS), is a specialized membrane that concentrically wraps around axons, forming insulating sheaths that support action potential conduction. Myelin reduces axonal membrane capacitance, increases membrane resistance, and facilitates the clustering of voltage-gated Na^+^ channels to the short unmyelinated gaps between myelin sheaths, termed nodes of Ranvier. This organization enables the regeneration of action potentials specifically at nodes of Ranvier, in a process termed saltatory conduction [1,2], which significantly increases conduction velocity compared with unmyelinated axons of the same diameter [3]. Myelination restricts membrane depolarization, and subsequent energy-demanding repolarization, from the entire axonal membrane to the nodes of Ranvier. In invertebrates, which lack compact myelin, conduction velocity can only be increased through enlarging axon diameter. Consequently, the appearance of myelin in vertebrates has provided a space-saving and energy-efficient alternative which has allowed the evolution of complex vertebrate nervous systems [4,5]. In recent years, evidence for additional roles of myelination in supporting axonal metabolism and integrity has emerged [6,7]. Although the underlying mechanisms are not yet fully understood, this includes the regulated transfer of metabolic substrates and antioxidant support to the axon; as well as boosting axonal mitochondrial function and ion buffering [7–12]. Indeed, the relatively sparse myelination of some axons, such as those of neocortical inhibitory interneurons, is not predicted to significantly modulate conduction speed, and may instead serve to support axonal function through such mechanisms [13].

The extent of myelination along an axon significantly impacts both axonal excitability and conduction velocity [3,14,15], and thus the timing of neurotransmission. At a circuit level, information is encoded in the timing of action potential arrival. For example, the synchronous arrival of impulses on a common target is central to the process of synaptic strengthening [16]. Such forms of plasticity require millisecond precision in the arrival of action potentials. Therefore, it follows that changes to myelin could modulate synaptic plasticity, and thus circuit function [17].

Myelination begins around birth and continues throughout life, with the most significant increases occurring during childhood and adolescence [18–20]. Longitudinal *in vivo* imaging of the mouse CNS showed that new myelin can be generated via the differentiation of new oligodendrocytes from precursors present throughout the brain (oligodendrocyte precursor cells, OPCs) [21] or through the remodelling of existing myelin, even in the adult CNS [22–24]. Over the last decade, characterizing the myelination profiles of individual axons in adulthood revealed significant variation in sheath length, number, thickness, and distribution. Some axons can have relatively sparse and discontinuous myelination, in a perhaps surprising departure from the textbook description of a uniformly myelinated axon [25–27]. This heterogeneity implies that myelination is a locally refined process, rather than a simple binary of being present or absent. Over the life-course, it now seems clear that new myelin sheaths can be added to previously unmyelinated regions of axons [23,26]. Once formed, the majority of new myelin internodes remain stable over time but can exhibit some degree of remodelling [23,26]. It is yet to be determined to what extent *de novo* myelination versus remodelling of existing myelin occurs in different CNS regions, developmental stages or physiological conditions. Critically, recent studies have revealed a significant role for new myelin formation in learning and cognition, both in development [28] and adulthood [29–31]. The underlying mechanisms are still unclear, but *de novo* myelination could modulate synaptic plasticity to strengthen behaviourally relevant circuits. Overall, the recent studies on myelin dynamics suggest it is a plastic rather than static structure, that retains the ability to be added to and remodelled continuously throughout life.

The importance of appropriate myelin development and maintenance in CNS circuits is underscored by the severe consequences associated with myelin disruption. Animal models of CNS hypomyelination or dysmyelination exhibit disrupted action potential conduction velocity and circuit-specific behavioural impairments [32–34]. Additionally, myelin loss or disruption is linked with a number of neurodevelopmental, neurodegenerative and neuropsychiatric disorders –notably multiple sclerosis [35], but also leukodystrophies [36], autism spectrum disorder [37,38], amyotrophic lateral sclerosis [39], schizophrenia [40], among others. Enhancing the production of new myelin may in fact be a promising treatment strategy not only for demyelinating disorders such as multiple sclerosis [35,41], but also for neurodegenerative conditions [29], hypoxia-related white matter injuries [42,43], and potentially even chemotherapy-associated cognitive impairment [44]. Therefore, elucidating the mechanisms by which myelin is dynamically regulated in the healthy nervous system is fundamental to understand how it might go awry, and might be repaired, in a disease context.

Various intrinsic and extrinsic factors dynamically regulate myelination by modulating oligodendrocyte lineage cell development and/or potentially acting directly on existing myelin. Excellent reviews have covered the factors influencing oligodendrocyte generation, for example [45,46]. Here, we will focus on studies addressing the impact of one extrinsic factor, neuronal activity, in regulating CNS myelination directly.

## The importance of activity-regulated myelination for circuit function

The concept that neuronal activity influences myelination is not new – Gyllensten and Malmfors demonstrated in 1963 that animals reared in darkness had reduced myelination of the optic nerve [47], a finding supported by subsequent sensory deprivation studies [48–51]. Tasks of motor and spatial learning, spatial working memory, fear memory consolidation, and sensory enrichment in rodents increase oligodendrogenesis, *de novo* myelination, and sheath remodelling [22,26,30,31,52–57]. These findings are supported by diffusion tensor imaging studies in humans showing that learning novel motor tasks coincides with structural white matter tract changes [58,59] thought to primarily reflect alterations to myelinated axons [60]. As a whole, these studies indicate that certain forms of brain experience induce *de novo* myelination or remodelling of existing myelin, but cannot distinguish between a primary effect directly on myelin or an indirect effect from regulation of oligodendrocyte lineage progression.

In addition to manipulation of environmental experience, direct experimental manipulation of electrical activity in specific neurons has also provided evidence that activity influences oligodendrocyte development and myelination. Seminal studies in the developing rodent optic nerve blocked action potentials by injection of tetrodotoxin, and observed a significant decrease in OPCs, oligodendrocytes and myelin [61,62]. Since then, researchers have also exploited technological developments to address this question with refined neuron-specific genetic manipulations. For example, optogenetic stimulation of neurons in the mouse premotor cortex increased myelin thickness along their axons [63]. In the somatosensory cortex, chemogenetic activation biased the targeting of myelin towards stimulated axons and increased their myelin thickness [64]. Furthermore, chemogenetic stimulation of medial prefrontal inhibitory neurons increased the number of myelin sheaths along their axons, although this was partly due to increased arborization of the axons themselves [65]. More recently, transcranial magnetic stimulation was reported to increase the thickness of cortical myelinated axons without affecting myelin sheath length [66]. Unlike in optogenetic and chemogenetic studies above, this was due to an increase in the space between the axon and overlying myelin, termed the peri-axonal space [66]. Moreover, seminal studies used the zebrafish model to enable live imaging of myelin dynamics *in vivo* following constitutive manipulations of neuronal firing. Dampening action potential firing reduced oligodendrocyte and myelin sheath number, leading to fewer axons being selected for myelination [67,68]; while increasing network or single-neuron activity boosted oligodendrocyte and sheath numbers and length [67,69].

Overall, these studies made clear that changing neuronal firing rates regulates myelin structure across vertebrate species, but the underlying signal that conveyed activity levels to oligodendrocytes *in vivo* remained (and remains) not entirely understood. A hint came from zebrafish studies that used genetically encoded tetanus or botulinum neurotoxins to prevent activity-dependent synaptic vesicle fusion in individual neurons. These studies revealed that globally blocking vesicle fusion prevented the increase in myelin following network activity enhancement and, when used to silence individual neurons, reduced the growth and stable maintenance of newly-formed myelin sheaths [68–70]. Thus, activity-regulated vesicle fusion and, presumably, associated secretion of vesicle cargoes is at least one of the signals that mediate activity-regulated myelination, which we will discuss further next.

These studies collectively highlight how alterations in neuronal activity can influence multiple aspects of myelination: myelin sheath number, length, distribution, thickness and the axon–myelin interface. Even seemingly minor changes in any one of these parameters could potentially regulate conduction velocity [3,14,15] or metabolic support to modulate circuit output and brain function.

**Figure 1:**
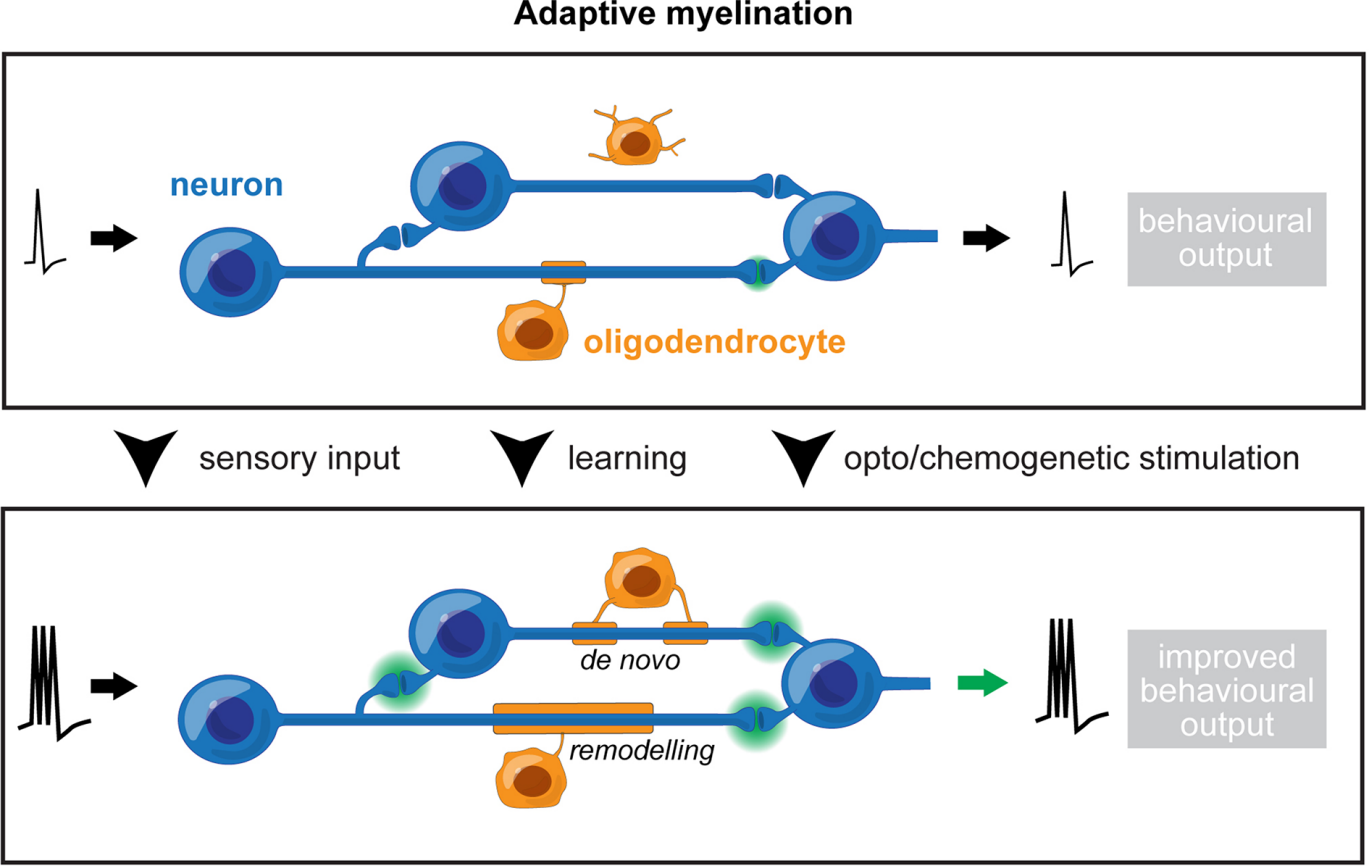
Adaptive myelination as a form of neural circuit plasticity Changes in neuronal activity induce remodelling of existing myelin or *de novo* production of new myelin sheaths to regulate axonal conduction of action potentials and the functional output of neural circuits.

Therefore, the concept of ‘adaptive myelination’ as a form of neural circuit plasticity has gained traction over recent years. In adaptive myelination, circuits alter their myelin in response to external stimuli, enabling adaptation of their physiology and of the corresponding behavioural output to respond to lived experience (Figure 1). Indeed, a small number of studies have linked activity-induced changes in myelin to improved functional outcomes [31,44,63]. For example, Gibson et al. demonstrated that the unilateral optogenetic stimulation of the motor cortex which increased myelin thickness was associated with improved motor performance in the corresponding limb [63]. Perturbing these activity-dependent myelin changes prevented behavioural improvements, supporting the premise of adaptive myelination [63]. Subsequently, the same team demonstrated that blocking activity-dependent myelin changes also impaired novel object recognition [44]. Moreover, recent research has shown that auditory stimulation selectively increased myelin thickness on active axons, while the use of earplugs to block auditory input prevented this adaptive myelination response and impaired auditory temporal processing [71]. Additionally, spatial learning has been linked to an increase in mature oligodendrocytes both during and immediately after training in a water maze task, which correlated with an increased number of myelinated axons in the corpus callosum [31]. When the formation of new oligodendrocytes was prevented either during or following training, animals could still learn the task but showed impairments in memory recall, demonstrating the importance of *de novo* myelination for spatial memory consolidation [31]. Overall, these findings suggest that sensory processing, motor functions, as well as cognitive behaviours, can be influenced by myelin adaptations in response to experience [44].

These studies underscore the importance of activity-regulated myelination in modulating the actual functional output of circuits. While they highlight regional changes to myelination, e.g. by tissue-level analysis of myelin or oligodendrocytes, or cross-sectional electron microscopy of whole axonal tracts, what has remained unclear are the precise subcellular and molecular mechanisms by which individual neurons convey activity information specifically to myelin sheaths associated with their axons.

## How are neuronal activity levels conveyed to oligodendrocytes?

**Figure 2:**
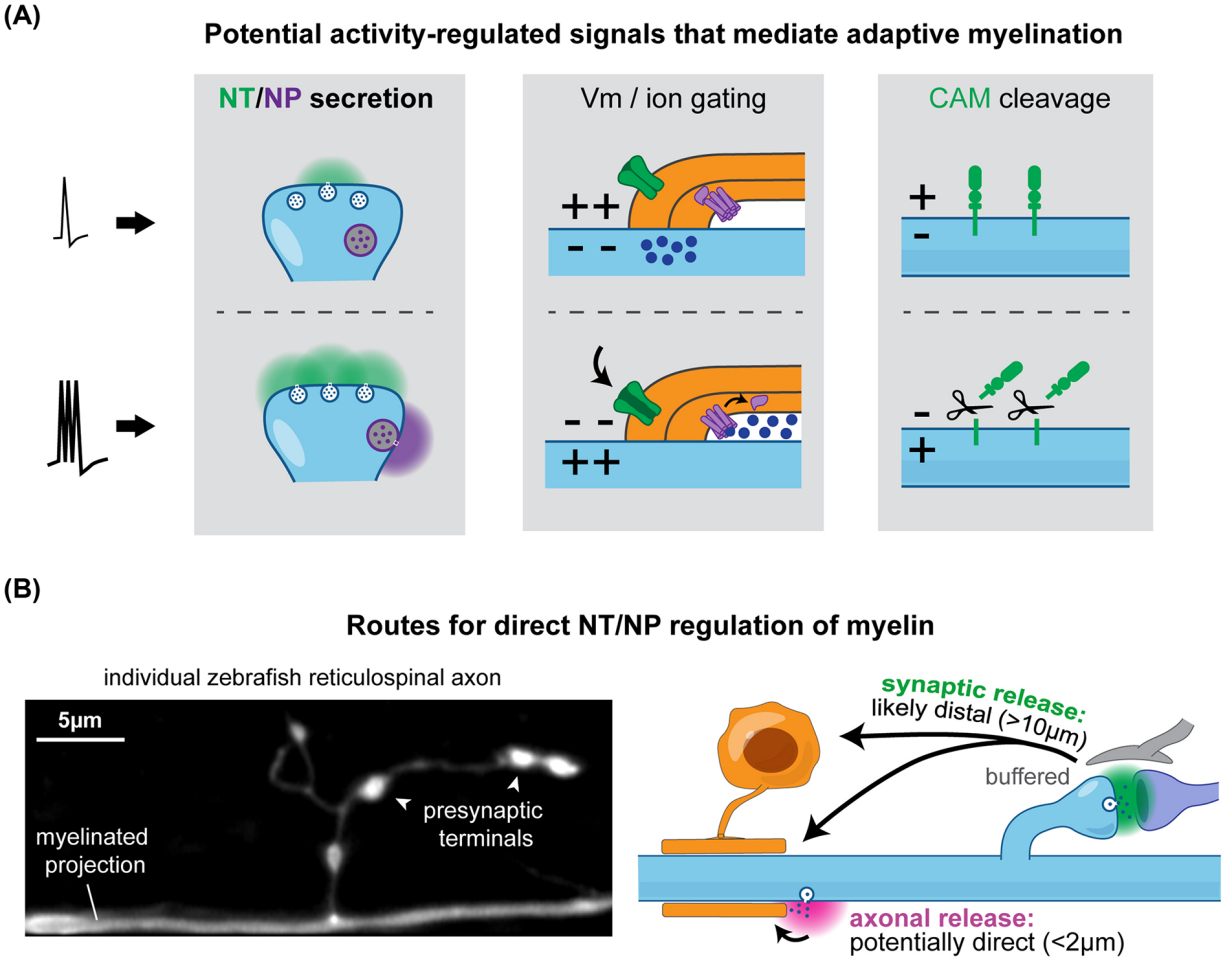
Potential mechanisms by which neuronal activity may regulate myelination (**A**) Various candidate activity-regulated axonal signals. (**B**) Regulation of myelination by secreted signals is likely direct from local axonal release, since synaptic release can be distal from myelin and highly buffered.

Several molecular and cellular mechanisms can be envisaged by which individual neurons could communicate activity information to nearby oligodendrocytes to induce myelin adaptations (Figure 2A). A key consideration is whether adaptive changes are specifically induced in the myelin sheaths associated with the individual axon of the stimulated neuron. Clearly, the promotion of oligodendrocyte-lineage cell proliferation and progression following certain stimulation paradigms affects multiple cells and may result in increased myelination in a whole axonal tract or region, but not with single-axon specificity. For example, when Gibson and colleagues optogenetically stimulated cortical neuronal tracts, they observed increased oligodendrocyte-lineage cell number and subsequently increased myelin thickness in many callosal axons on the stimulated side [63]. In this case, it was not possible to ascertain if the myelin adaptations were restricted only to stimulated axons individually, or instead locally affected a whole cohort of neighbouring axons. Since this study, multiple lines of evidence have indicated an ‘autocrine’ mechanism whereby modulating activity of individual neurons specifically or preferentially affects their own myelin sheaths. For example, Bacmeister et al. recently reported changes in myelin sheath length and the addition of newly formed sheaths specifically to motor axons that were identified as being activated during the process of learning a motor task [56]. Further studies used sophisticated opto- and chemogenetic tools that allow for specific manipulation of activity in individual neurons and analysed myelination along their respective axons. For example, using the zebrafish model, several studies found that chemogenetic stimulation or abolishment of vesicle fusion in single neurons respectively increased or decreased the chances of their own axons becoming myelinated. This pro-myelination effect observed *in vivo* was not limited to axonal selection, as manipulating activity also regulated the extent to which an axon became myelinated, altering the number and length of myelin sheaths made along their own axons [68–70]. In mammals, Mitew and colleagues found that decreasing excitability in neurons by overexpressing the inward rectifying potassium channel Kir2.1 specifically reduced the likelihood that axons of Kir2.1-overexpressing somatosensory neurons became myelinated, while chemogenetically increasing activity using Gi-DREADDs increased myelination of Gi-DREADD-expressing axons specifically [64]. They also identified an increase in myelin thickness specifically in Gi-DREADD-expressing axons using immunogold electron microscopy. On the other hand, in a striking example of ‘paracrine’ activity-regulated myelination, Osso et al recently showed that forced swim stress stimulated secretion of the neuropeptide dynorphin by non-myelinated neurons in the striatum, promoting myelination not of their own axons, but of the larger axons of neighbouring dynorphin-negative neurons [72]. Nevertheless, most of these studies strongly suggest that where there is activity-regulated promotion of myelination per se, this occurs directly on a neuron’s own myelin sheaths. What are the activity-regulated cellular and molecular mechanisms by which an individual neuron specifically promotes either *de novo* myelination of its own axon by nearby pre-myelinating oligodendrocytes, or induce remodelling of its existing myelin sheaths *in vivo*?

### Activity-dependent secretion

One of the most well studied activity-regulated processes by which neurons communicate neuronal firing is through activity-dependent secretion of chemical messengers that act on a target cell or tissue. Depolarization of axon terminals triggers tightly regulated synaptic vesicle fusion, releasing neurotransmitters such as glutamate or γ-aminobutyric acid (GABA) into the synaptic cleft, from where they then bind postsynaptic receptors that regulate the activity of target cells. While most neurotransmitter is recycled or buffered by astrocytes, in certain conditions, such as high frequency firing, ‘spill-over’ from the synaptic cleft into nearby extra-synaptic regions can occur [73,74]. Furthermore, alongside synaptic vesicles, neuronal presynaptic terminals also contain large dense-core vesicles filled with neuropeptides and neurotrophins. These are also released in an activity-dependent manner, typically after high firing frequency [75]. Peptidergic signals such as Brain-derived neurotrophic factor (BDNF) [76], dynorphin and neuropeptide Y typically diffuse into broader areas than the synaptic cleft and bind G-protein coupled receptors in target cells. Oligodendrocyte-lineage cells express many neurotransmitter and peptidergic receptors [77] (discussed below), including high-affinity metabotropic receptors [78], which could sense even slight rises in ambient neurotransmitter/neuropeptide concentration, and could induce long-lasting cellular responses that ultimately regulate myelination, but the sequence of events from receptor activation and ending in *de novo* myelination or myelin remodelling remain to be fully dissected.

### Voltage/ion-dependent mechanisms

When the axolemma depolarizes and ion concentrations change around the axonal membrane, voltage/ion-gated channels or receptors in associated myelinating oligodendrocyte processes could initiate a signalling cascade promoting *de novo* ensheathment or sheath remodelling. Voltage-gated sodium, calcium and potassium channels are expressed in OPCs [79,80] and some persist in differentiated oligodendrocytes, such as the voltage-gated calcium channel Cav2.3 [81], and the inward-rectifying potassium channels, Kir4.1 [82,83] and Kir7.1 [84]. Activation of such channels or receptors could culminate in *de novo* myelination or myelin remodelling. Interestingly, Kir4.1 knock-out in oligodendrocytes did not overtly disrupt myelination, but compromised axonal ionic and metabolic homeostasis [9,12,85], suggesting that potassium signalling via this route may primarily enable oligodendroglia to acutely sense and support high axonal activity levels. Nevertheless, recent studies have also found functional hyperpolarization-activated cation HCN2 channels to be expressed in differentiated oligodendrocytes *in vivo* [86], and to be necessary for appropriate myelin sheath elongation [87]. Therefore, this signalling mode could also affect myelination per se, through yet to be fully characterized mechanisms.

### Activity-regulated modulation of adhesion

Axolemma depolarization could also trigger myelin formation or remodelling by inducing proteolytic cleavage of axon-tethered cell adhesion molecules to recruit nearby oligodendrocyte processes. For example, *neuregulin 1* (Nrg1) encodes a family of secreted or axon-tethered adhesion molecules that instruct myelination in the PNS through interaction with ErbB receptors expressed on myelinating glia [88–90], and which are also present in the CNS [91,92]. The extracellular domain of some Nrg1 molecules (subtypes I, II and IV) can be cleaved in response to neuronal activity [93–95]. Interestingly, a recent study in zebrafish found that Nrg1 type II produced by a subtype of unmyelinated sensory spinal neurons non-cell-autonomously promoted myelination of a nearby subtype of myelinated interneurons [96]. It will be important to determine if activity-dependent processing of Nrg1 or any other cell-adhesion molecules can induce responses in oligodendrocytes that are consistent with adaptive myelination *in vivo*.

### Additional indirect mechanisms

Activity levels may also be conveyed more indirectly to myelin, through other local physiological changes to axonal tracts. For example, direct or astrocyte-mediated signalling into blood vessels regulates local blood flow to match glucose and oxygen provision to local neuronal demand [97]. Oxygen tension is known to regulate OPC differentiation and postnatal myelination in mice [98]; and vascular signals such as endothelin can regulate how much myelin individual oligodendrocytes form, and mediate experience-driven changes to myelination in the prefrontal cortex following social isolation [99]. Furthermore, astrocytes closely associated with myelinated axons can additionally release ATP to the extracellular space, where it is converted to adenosine, which in turn signals back to the axon through A_2a_ receptors and HCN2 channels to regulate excitability and conduction velocity [100]. Astrocyte-released ATP/adenosine could also activate adenosine receptors present in oligodendrocyte-lineage cells [101,102] to regulate myelin plasticity. Another activity-driven process is the remodelling of the extracellular matrix (ECM), e.g. around synapses, to support their plasticity [103]. ECM molecules also coat myelinated axons, interleave with myelin sheaths and associate with nodes of Ranvier [104,105], so ECM remodelling could also locally regulate periods of myelin plasticity along axonal tracts. Moreover, activity-induced ion flows can also alter the physical properties of axon, e.g., by increasing axonal diameter [106–108], which by itself regulates myelination [51,55,109] but see [110]. Furthermore, sheath integrity can be compromised by activity-induced osmotic effects: Na-K-Cl cotransporter NKCC1 loss at the axon–myelin interface caused myelin swelling, which was resolved by dampening neuronal activity [111,112].

How these indirect pathways by which activity regulates myelination interact with the more direct signalling routes described above remains to be fully dissected *in vivo*.

## Evidence for activity-regulated secretion as a key mechanism

Activity-dependent neurotransmitter release onto myelinating oligodendrocytes has recently garnered significant attention. In differentiated neurons, activity-regulated secretion is a carefully controlled process that occurs at highly specialized presynaptic terminals in the axon. Presynaptic terminals contain an array of specific molecules, including cytomatrix, scaffolding, vesicle-associated and plasma membrane-tethered proteins, ion channels, sensors, and regulatory accessory proteins [113]. This conserved machinery ensures that the arrival of an action potential triggers a sequence of steps – neurotransmitter vesicle docking, priming, fusion, endocytosis, recycling and refilling – with millisecond and nanoscopic precision [114]. This machinery also enables modulation of neurotransmitter release through various forms of short and long-term presynaptic plasticity. Once released into the synaptic cleft, neurotransmitters bind to postsynaptic receptors to signal to downstream neurons. They are then metabolized by degradatory enzymes, or spill-over onto nearby structures, and are subsequently taken up by nearby transporter molecules. In this way, the lifetime of transmitters in the cleft is very short, in the order of milliseconds [115,116].

How can neurotransmitters released at these sites promote *de novo* myelination or sheath remodelling? *In vivo*, the myelinated axonal projection can be located at a long distance from the presynaptic terminals in the neuropil, especially in white matter tracts. For instance, in the zebrafish spinal cord, reticulospinal neurons have their soma located in the hindbrain and extend a main axonal projection along the entire length of the ventral spinal cord, which becomes myelinated. At intervals, collateral branches with a ramified morphology sprout off this main axonal projection, which remain unmyelinated, and form presynaptic terminals with downstream targets. These terminals are located potentially tens of micrometres away from the myelin sheaths (Figure 2B) (or even hundreds in the case of mammalian white matter). The number and length of myelin sheaths along reticulospinal axons is greatly reduced when individual neurons express neurotoxins to abolish vesicle fusion [69,70], indicating that in each reticulospinal neuron, synaptic vesicle cargo normally signals directly to oligodendrocyte processes or myelin sheaths associated specifically with its axon. Synaptically-released neurotransmitters may therefore have to diffuse over a significant distance to reach either existing myelin sheaths, oligodendrocyte processes or their soma to signal an increase in activity. Recent experimental and modelling studies suggest that certain CNS regions, such as the hippocampal neuropil, are in fact more permissible to short-range (up to 2 µm) glutamate spread than previously thought [73,117,118]. However, in most regions, astrocytes can surround synapses with their numerous, ramified fine processes packed with high-affinity neurotransmitter transporters, and thus greatly limit neurotransmitter diffusion even for extra-synaptic, spilled over neurotransmitters [119]. It therefore seems unlikely that a synapse-to-myelin or synapse-to-oligodendrocyte route can specifically and reliably promote myelination of individual axons *in vivo* (Figure 2B). Instead, a more localized mechanism may be needed to mediate activity-regulated myelination *in vivo*.

How can neurotransmitter release, generally considered to occur only at active zones of synapses, reach myelin? In fact, activity-regulated secretion from neurons can occur away from presynaptic terminals. For example, most peptidergic neurotransmitters are released in a non-synaptic manner [75,120–122]. Entire neuromodulatory populations, such as monoaminergic neurons, release neurotransmitters from axonal sites that do not form classical synaptic contacts [123–125] (although typically their axons are not myelinated [72,126]). The identification of such non-synaptic release has led to the concept of ‘volume’ or ‘wireless’ neurotransmission, whereby neurochemical messengers released onto the extracellular space and the cerebrospinal fluid diffuse over larger distances than synapses to exert their effects over a broader anatomical reach (reviewed in [127–132]). Crucially, however, both historical and emerging studies indicate that even neurons which release classic fast amino acid neurotransmitters such as glutamate can do so at axonal hotspots distant from presynaptic active zones.

## What is the evidence for axonal neurotransmitter release?

‘Ectopic’ fusion of synaptic vesicles at non-synaptic axonal sites has been described over four decades ago, in an electron microscopy study of the neuromuscular junction that also described a stark increase in such ectopic fusion in conditions of high activity [133]. Subsequently, *in vitro*, cultured Xenopus spinal cord neurons were shown to release acetylcholine in an activity-dependent manner along their developing axonal projections prior to synapse formation by recording membrane currents from myocytes made to contact axons [134–136]. These findings were supported by studies of cultured mammalian hippocampal neurons, whose developing axons were also shown to exhibit synaptic vesicle recycling independently of synaptic terminals, as detected by uptake of fluorescently-labelled synaptic vesicles [137–139]. These *in vitro* studies, while showing that axonal neurotransmission was in principle possible in very distinct systems, did not necessarily indicate it was a physiological phenomenon. Supporting *in vivo* evidence for non-synaptic neurotransmission came from ultrastructural studies showing the presence of synaptic vesicles docked at non-synaptic sites in frog saccular hair cells [140] and in the chick ciliary ganglion [141], as well as imaging studies showing some synaptic vesicle fusion outside of active zones in retinal bipolar cells [142,143]. Clearer evidence came from *ex vivo* functional studies in the cerebellum, where ectopic glutamate release sites in climbing fibers were shown to specifically mediate communication with Bergmann glial cells, separately from their well characterized synaptic connections made with Purkinje cells [144–146]. Calcium-dependent synaptic vesicle exocytosis was also shown to take place in axonal hotspots distinct from presynaptic terminals in mature glutamatergic neurons *ex vivo*, in hippocampal slices [147]. Additionally, other classical neurotransmitters were found to be released at discrete sites along axons in a physiologically relevant neuron-glia signaling mechanism. For instance, electrical stimulation of neurons in *ex vivo* preparations could elicit non-synaptic neurotransmission mediated by ATP in dorsal root ganglia [148] and in the olfactory bulb [149]. Functional GABA_A_ receptors are also present in axonal non-synaptic membrane of mossy fibres of granule cells of the dentate gyrus, suggesting a non-synaptic form of neurotransmission [150].

**Figure 3:**
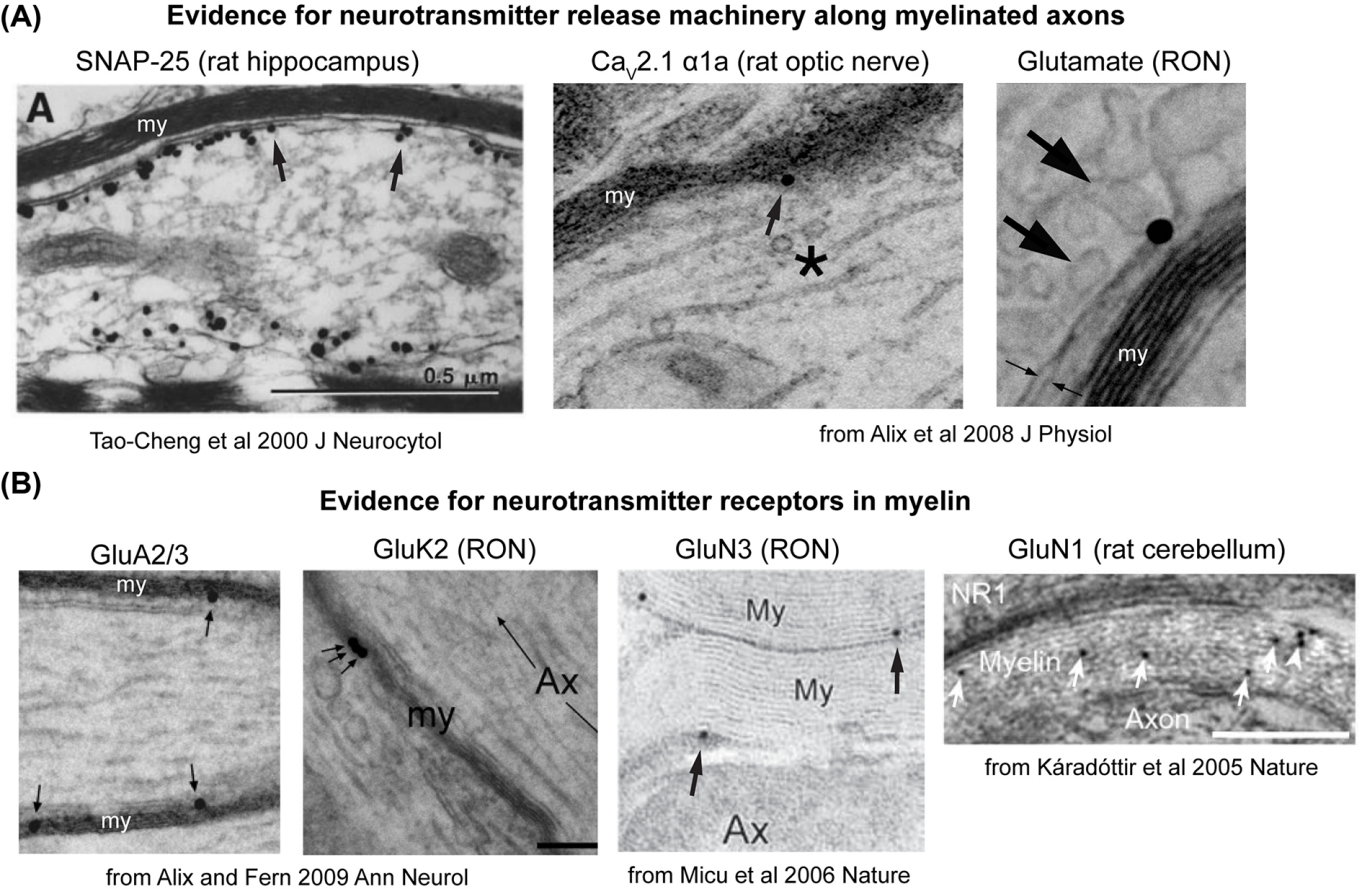
*In vivo* evidence for endogenous neurotransmission proteins non-synaptically localized along myelinated axons (**A**) Immuno-gold electron microscopy evidence for vesicle fusion machinery including SNARE protein SNAP-25, voltage-gated calcium channel subunits Ca_v_2.1 and glutamate along the axonal membrane under the myelin sheaths (‘my’), indicated by arrows. Asterisk in middle panel indicates vesiculotubular elements. (**B**) Immuno-gold electron microscopy evidence for GluA (AMPA), GluK (kainate) and GluN (NMDA) glutamate receptor subunits present within myelin sheaths (‘my’), indicated by arrows. ‘RON’ denotes rat optic nerve, ‘my’ denotes myelin sheaths, ‘Ax’ denotes axon. Adapted with permission from references 153, 157, 194, 195 and 197.

Additional supporting evidence for axonal neurotransmitter release comes from studies on the localization of vesicle fusion machinery that is typically thought of as ‘synaptic’, such as the SNARE proteins that fuse vesicle and axonal membranes, or the voltage-gated Ca^2+^ channels through which Ca^2+^ enters the presynaptic terminals following an action potential. Immunolabelling techniques for light and electron microscopy have identified the presence of SNARE proteins Syntaxin1 and SNAP25, as well as the α1A subunit of P/Q-type Ca^2+^ channels (CACNA1A) and glutamate, along non-synaptic regions of the axonal membrane *in vivo* (Figure 3A), including in the myelinated axolemma in the optic nerve, cortex, brainstem and hippocampus [135,151–155]. Since cell-free assays have shown that the minimal requirement for membrane fusion is the presence of a Syntaxin and a SNAP25 family protein on one membrane and a VAMP family protein on the other [156,157], these localization studies imply that the required machinery for neurotransmitter vesicles to fuse along the axon is present non-synaptically along its length. Indeed, in myelinated optic nerve axons, vesiculotubular profiles positive for glutamate immunoreactivity were observed in the process of docking and fusing with myelinated axolemma itself [151,158].

It is important to note that key seminal studies in mammals revealed clear glutamate release from unmyelinated callosal axons *in vivo*, away from classical neuron-to-neuron synapses, occurring instead at discrete synapses formed with OPCs. These axon–OPC connections have been identified throughout the CNS and shown to be bona-fide glutamatergic or GABAergic synapses at a molecular, electrophysiological and ultrastructural level [80,159–171]. They are hypothesized to regulate OPC proliferation and differentiation, which has recently been addressed in studies that manipulated postsynaptic molecules within OPCs: e.g. glutamatergic signaling through OPC AMPA receptors promoted cell survival [166], and GABA receptor signaling regulates targeting and size of myelinated axonal domains in interneurons [167,168,172]. In zebrafish, targeting postsynaptic scaffolds or cell-adhesion molecules in OPCs reduced oligodendrocyte number and myelination [169]. However, it is important to note that all of the observed myelin effects may be secondary to primary changes in oligodendrogenesis; indeed, axon–OPC synapses disappear as OPCs differentiate [80,170]. OPCs are also increasingly appreciated to perform a range of oligodendrogenesis-independent functions in the CNS [171,173], which could be the primary target of axon-OPC synaptic signaling. Given this mode of communication is legitimately synaptic and its properties have been extensively reviewed e.g [174,175]. Here, we focus instead on the *in vivo* evidence for a non-synaptic mode of direct neurotransmitter signaling between axons and myelin sheaths in response to neuronal activity.

Accumulating evidence indicates that activity-regulated neurotransmitter release can occur along myelinated axons and that it regulates myelin formation and growth. In a simplified *in vitro* system, vesicular release from neurons stimulated myelination, but no synaptic axon-oligodendrocyte junctions were identified [176]. In a separate *ex vivo* study, axonal release of glutamate was reported following electrical stimulation of myelinated rodent optic nerves, which subsequently elicited Ca^2+^ rises within the cytosolic compartment of the overlying myelin [158]. Electrophysiological recordings offer high temporal resolution for studying spontaneous and evoked neurotransmitter release mechanisms. However, the limited axonal accessibility due to the presence of myelin poses technical challenges for direct patch-clamp recording from myelinated axons [177]. Additionally, the invasive tissue preparation process, along with maintenance in artificial conditions, disrupts once-intact circuits and may not faithfully represent the *in vivo* environment. Moreover, techniques such as electron microscopy provide only a static snapshot of what is a dynamic process. What has remained unclear until recently is how relevant non-synaptic, axonal neurotransmitter release is in myelinated neural circuits *in vivo*. This means determining how frequent axonal vesicle fusion is along the length of individual intact axons *in vivo*, and how it is spatiotemporally related to the process of myelination.

## A new approach to study axonal neurotransmission *in vivo*

To accurately understand and quantify the dynamics of axonal vesicle fusion *in vivo* requires a system in which intact neural circuits can be imaged in real-time, in an entirely non-invasive manner, along the lengths of individually labelled axons (as opposed to a cross-sectional snapshot analysis of whole axonal tracts). While this has been technically challenging to achieve in rodent models, the suitability of zebrafish for live imaging makes it an ideal system to investigate axonal neurotransmission. The genetic tractability for single-cell labelling and adaptation of fluorescent exocytosis reporters enables monitoring of vesicular activity along entire axons [69,178]. Combined with concomitant labelling of all myelin sheaths along individual axons [70] and imaged at subcellular resolution, the zebrafish system enables determining the spatiotemporal relationship of axonal vesicle fusion dynamics with myelination. Furthermore, simple loss or gain-of-function genetic manipulations are available to test the causal relationships between these two processes [179].

Zebrafish studies first showed that neuronal activity regulated myelination via synaptic vesicle fusion *in vivo*. Mensch and colleagues showed that globally blocking synaptic vesicle fusion in whole embryos using Tetanus toxin (TeNT) caused spinal cord oligodendrocytes to produce fewer myelin sheaths [67], and also blocked an increase in sheath number that followed enhancement of network activity. In parallel, Hines and colleagues showed that TeNT-EGFP expression in individual spinal neurons reduced their selection for myelination, as well as nascent sheath length when they did become myelinated [68]. By transgenic overexpression of synaptic vesicle markers (e.g. Synaptophysin-EGFP) in single neurons, Hines et al and subsequently Hughes et al. [178] quantified labelled puncta along the axon and observed higher densities at sites of oligodendrocyte ensheathment along the main axonal projection that becomes myelinated. This initial ensheathment of the axon was not affected by blocking vesicle fusion with TeNT, a finding confirmed by a recent study showing that oligodendrocytes dynamically and repeatedly ensheath the same axons multiple times, at a rate that was unaffected by global dampening of neuronal activity [180]. Instead, time-lapse imaging showed that nascent sheaths retracted more frequently in TeNT-expressing animals [68]. These results suggested a model of preferential maintenance of nascent sheaths on more active axons, rather than a preferential initial ensheathment of active axons. Subsequent studies showed that synaptic vesicle fusion-dependent regulation of myelination was a property of some, but not all neuronal subtypes [70]; and that oligodendrocytes express ‘postsynaptic’ molecules that could mediate a cellular response to the synaptic vesicle cargo [178].

To definitively resolve the spatiotemporal relationship between neurotransmitter vesicle fusion and myelination along individual cells, we recently adopted a reporter of synaptic vesicle exocytosis with enhanced signal-to-noise ratio [181], SypHy, for zebrafish expression. This reporter, coupled with highly resolved timelapse microscopy, enabled real-time live-imaging of vesicle fusion in individual myelinated axons and their unmyelinated, synapse-bearing collateral branches [69]. Remarkably, SypHy events consistent with known kinetics of exocytotic events occurred with similar frequency, amplitude and duration in collateral branches and along the myelinated axonal projection, in which canonical presynaptic terminals have not been identified. SypHy events co-localized with the glutamatergic marker Vglut1 and were blocked by botulinum neurotoxin, validating these events as bona-fide ‘synaptic’ vesicle exocytosis at non-synaptic axonal hotspots, during reticulospinal neuron myelination [69].

To concomitantly analyse myelination, we co-expressed an axonal reporter that enables accurate determination of sheath number, length and position along individual axons [70]. Neurons not yet myelinated had few SypHy events, suggesting that the onset of myelination induces axonal vesicle fusion. Indeed, specifically disrupting myelination using a zebrafish mutant for *myrf* [32], which encodes a transcription factor essential for terminal oligodendrocyte differentiation [182–184], significantly reduced axonal but not synaptic vesicle fusion. This indicates that myelination promotes axonal vesicle fusion, and it will be interesting to dissect the underlying mechanisms by which this occurs. In axons undergoing myelination, axonal vesicle fusion was three times more frequent in unmyelinated regions than in myelinated regions of the same axon. This surprising observation contrasted with previous studies suggesting an enrichment of synaptic vesicles at sites of ensheathment [68,178] but it is worth noting the higher subcellular resolution afforded by our SypHy and myelination reporters, and that earlier studies were capturing only static snapshots of constitutively labelled synaptic vesicles, rather than exocytosis dynamics. Closer examination indicated that SypHy events were not uniformly distributed along the unmyelinated axonal segments, but were enriched at hotspots adjacent to myelin sheaths. As these are the regions sheaths grow into, axonal vesicle fusion is therefore suitably placed to regulate myelin production. At least some hotspots colocalized with a tagged reporter of the zebrafish orthologue of neuronal Neurofascin (NF186), a cell-adhesion molecule present at nodes of Ranvier [69,185,186]. This confirms that axonal hotspots of vesicle fusion bear hallmarks of nodal domains. In fact, in peripheral myelinated axons, ion channels and NF186 are thought to be delivered by vesicular transport to mature nodes [187]. Thus, in principle, vesicle fusion machinery is present at nodal hotspots in myelinated axons to deliver transmembrane proteins to the axolemma, and it is possible that this machinery could also facilitate the capture, docking and fusion of neurotransmitter vesicles. It will be interesting to dissect exactly what ‘presynaptic’ machinery is present at these sites and how it compares to synaptic terminals (see ‘Final remarks and perspectives’).

To temporally relate axonal vesicle fusion and the rate of myelin formation and growth, we reimaged myelination several hours after imaging vesicle fusion. This analysis revealed that the frequency of vesicle fusion adjacent to nascent sheaths positively correlated with sheath growth. Perturbing vesicle fusion with BoNTB expression indeed reduced the stability of nascent sheaths, such that they were more likely to retract than control axons, and also reduced their growth rate. Conversely, stimulating neuronal activity through a chemogenetic strategy over the initial two days of myelination increased sheath elongation. Indeed, axonal SypHy activity was increased acutely by the chemogenetic manipulation that promoted sheath growth in reticulospinal neurons, indicating that axonal vesicle fusion is at least partly activity-driven [69]. It will be important to determine the exact link between action potentials and vesicle fusion at axonal hotspots, to determine how it compares to the highly specialized active zones at presynaptic terminals, and define the conditions in which neurons engage this mode of signalling.

Overall, these experiments indicate that local axonal vesicle fusion can directly regulate the stability and growth of myelin sheaths. It is important to note that these studies examined vesicular transport and fusion only, not the vesicular cargo itself, the presumed signal. Furthermore, they employed transgenic overexpression methodologies, and it will be important to validate these observations and working model using strategies that report the physiological dynamics of endogenous neurotransmitters.

## The glial perspective: sensing neurotransmitters in myelin

How might oligodendrocyte-lineage cells sense and respond to axonal neurotransmission *in vivo*? Transcriptomic and electrophysiological evidence suggests oligodendrocyte lineage cells acquire a range of ion channels and neurotransmitter receptors making them sensitive to activity-regulated secretion from neurons [188–191]. However, relatively few papers have investigated at the protein level and with subcellular resolution whether expression remains after the onset of myelination, and if neurotransmitter receptors are present on the myelin membrane itself. Presumably, receptors localized on myelin would provide a direct signalling mechanism by which individual sheaths could sense and respond to changing levels of neurotransmitter released from axons. Immunocytochemical localization of the main ionotropic glutamate receptors, AMPA and NMDA, showed expression on mature oligodendrocytes and, to some degree, on the myelin itself [192–197]. NMDA receptors were found to be preferentially localized on myelinating processes, whereas AMPA receptors appeared to be more concentrated on the soma of oligodendrocytes [197]. Ultrastructural analysis using immunogold labelling (Figure 3B) confirmed localization of AMPA and NMDA subunits also within the compacted myelin [192,193,195]. Subunits for both receptors could be observed on the inner myelin layer facing the axolemma [192,195], whereas NMDA receptor subunits were additionally observed in-between layers and on the outermost myelin layer [193,195]. The presence of GluR subunits on the myelin layers could enable existing sheaths to sense and respond to glutamate released either from the underlying axon facing the inner myelin layer or externally from neighbouring axons. Despite a small number of papers reporting the expression of metabotropic glutamate receptors on oligodendrocytes *in vitro* [198,199] and in the post-natal rat brain [200], it is yet to be determined to what extent these receptors could mediate a functional form of signalling between neurons and myelinating oligodendrocytes. In agreement with the premise that axons communicate with myelin via secreted neurotransmitters, oligodendrocytes also express the neurotransmitter receptor-associated scaffolding protein PSD95 [178,189], as well as transporters of glutamate and the necessary machinery for glutamate metabolism [201,202], suggesting that like at synapses, oligodendrocytes can clear excess extracellular glutamate [191,203,204]. Collectively, these studies suggest that the myelin surrounding axons could be directly responsive to neuronal activity. However, it remains unclear whether myelin is generally responsive to activity along its length or only at specialized regions or domains in close apposition to sites of axonal vesicular release adjacent to the growing edges of myelin sheaths [69]. These paranodal sites of axo-glial contact contain several cell-adhesion proteins that anchor the myelin to the underlying axon. Expression of the glial isoform of Neurofascin forms a complex with axonally-expressed Contactin and Contactin-associated protein (Caspr) to form septate-like junctions, which are critical for the stability of nodes and act as physical barriers preventing diffusion of nodal components. Loss of these cell-adhesion proteins results in defective attachment of the myelin to the axon, impedes oligodendrocyte process migration and reduces sheath length [205–208], highlighting the importance of these domains for myelin growth. Therefore, it will be important to study neurotransmitter signalling at individual paranodes during activity-regulated myelination, as they may be a parsimonious locus to regulate sheath length.

What could be the potential downstream mechanisms following neurotransmitter receptor activation on myelin? During myelination, individual myelin sheaths exhibit local Ca^2+^ transients [209], partly driven by neuronal activity, and which can have distinct properties predictive of myelin sheath fate *in vivo* [210,211]. For example, high-amplitude, long duration Ca^2+^ transients were reported to precede myelin sheath retraction through the activation of the Ca^2+^-dependent protease calpain; whereas high-frequency Ca^2+^ activity in established sheaths correlated with faster elongation [210]. Genetically attenuating Ca^2+^ activity in oligodendrocytes had no effect on myelin thickness or the number of sheaths made, but significantly reduced sheath length through an actin-dependent mechanism [212]. Therefore, Ca^2+^ signalling in oligodendrocytes is a likely candidate to mediate sheath growth or retraction in response to changing levels of neuronal activity through modulating the actin cytoskeleton. Future studies will be required to dissect the exact molecular mechanisms controlling Ca^2+^-mediated and Ca^2+^-independent myelin remodelling or recruitment.

## What next in neurotransmitter-regulated myelination?

**Figure 4:**
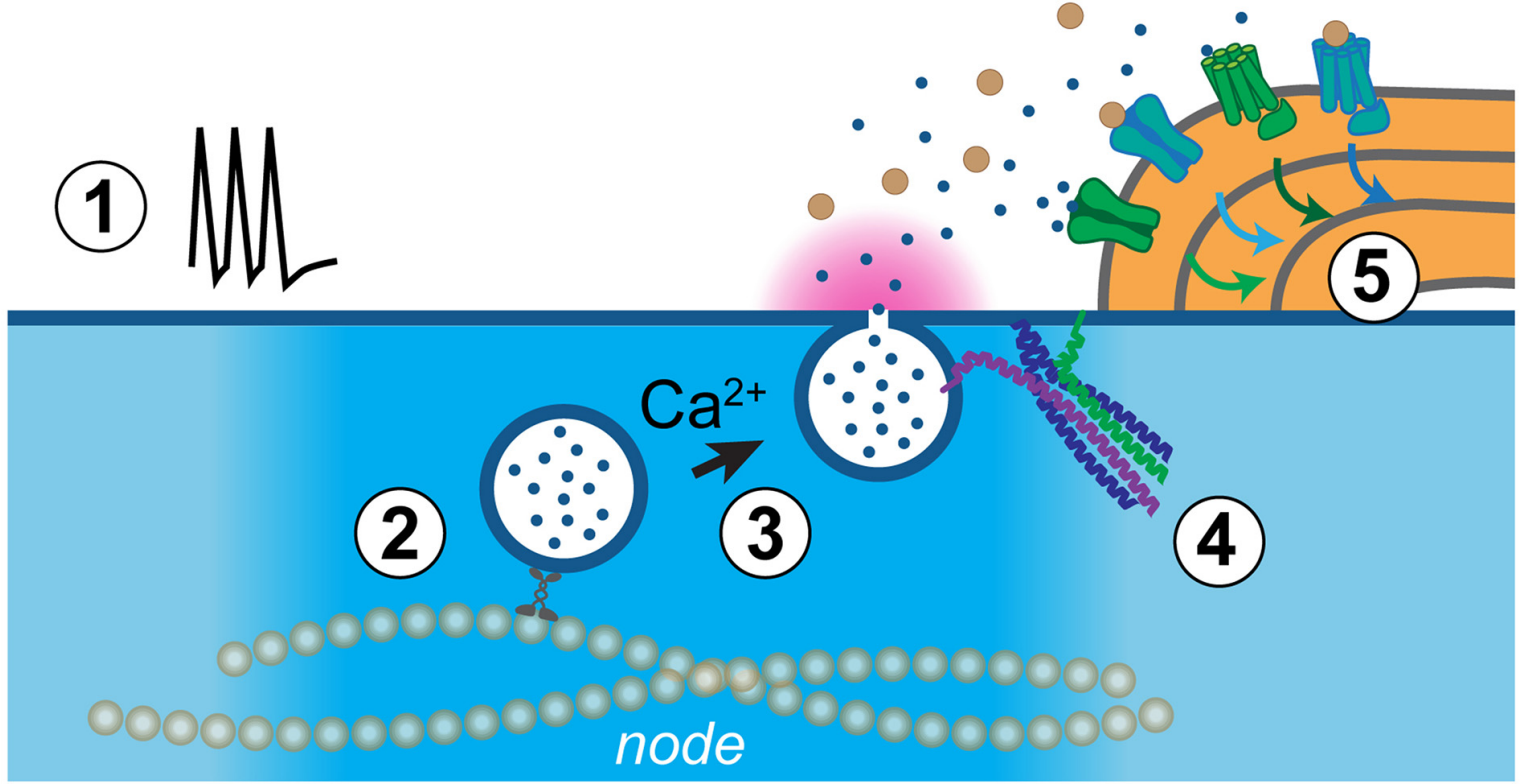
Outstanding mechanistic questions on axonal neurotransmission Five outstanding mechanistic questions on axonal neurotransmitter release and its regulation of myelin sheaths *in vivo*. See section "What next in neurotransmitter-regulated myelination?" for more details.

A number of outstanding questions need to be addressed to dissect the cellular and molecular mechanisms underpinning axonal neurotransmission to myelin (Figure 4):

### What levels of activity drive axonal vesicle fusion?

Axonal vesicle fusion is at least partly driven by neuronal activity levels, but whether this occurs synchronously and with high fidelity for each action potential, as at synapses, is unclear. Potentially, only certain levels or patterns of activity trigger axonal release, such as when neurons fire at high frequency. Elucidating the conditions in which individual neurons drive axonal versus synaptic neurotransmitter release is a key first step to understanding the roles of this signalling mode.

### Which nodal specializations facilitate vesicular trafficking and release at axonal hotspots?

With myelination, axons become polarized into distinct subdomains, each with unique molecular infrastructure – e.g. cytoskeletal and transmembrane specializations – which may interact with vesicle transport mechanisms and support vesicle fusion differentially. For example, in hippocampal neurons with en passant synapses, differential affinity of the synaptic vesicle transport complex to local microtubule modifications facilitate delivery at axonal varicosities [213,214]. In some GABAergic neuronal subtypes, nodes of Ranvier, which coincide with axonal release hotspots, are induced by molecular cues (e.g. contactin1) secreted from oligodendrocyte-lineage cells prior to myelination [215]. Therefore, it will be essential to correlate the spatiotemporal distribution of release hotspots with the emergence of endogenous axonal subdomains across neuronal subtypes, and to fully elucidate how these domains are specified *in vivo*.

### How does axonal Ca^2+^ control axonal vesicle fusion?

Vesicle fusion is tightly regulated by calcium activity at synapses, but whether the same regulation takes place at axonal hotspots is unknown. Differences in the underlying machinery could explain different properties of axonal versus synaptic release. For example, in mouse hippocampal neurons, extrasynaptic vesicle fusion occurs in an asynchronous/delayed manner [147]. Revealing these mechanisms may elucidate whether there are axonal-specific mechanisms of vesicle fusion, which would provide a molecular entry-point to conditionally target this process specifically, without disrupting synaptic transmission, and determine its impact in circuit function.

### Which SNAREs execute axonal vesicle fusion?

At synapses, an array of highly specialized SNARE isoforms mediates synaptic vesicle exocytosis and couples it to the arrival of action potentials with millisecond precision. The identity of the SNARE isoforms that execute axonal vesicle fusion is unclear. Axonal vesicle fusion is blocked by botulinum neurotoxin B (BoNT-B), but which of the BoNT-B-sensitive Vamp1/2/3 homologues is present at axonally-released vesicles, and which SNAP25 and Syntaxin homologues it binds to drive fusion with the axonal membrane is, as of yet, unknown. Multiple homologues exist in each SNARE family, which mediate exocytosis in various cellular contexts (e.g. different vesicles or subcellular locations), and some are poorly characterized *in vivo*. SNAP25 and Syntaxin1 themselves are not exclusively located at presynaptic terminals, so it will be important to determine whether these SNAREs are also endogenously expressed along those axons that exhibit vesicle fusion.

### How are diverse activity-regulated signals orchestrated to regulate adaptive myelination?

Neuronal activity affects multiple aspects of oligodendrocyte development and of myelination. While here we focused on direct regulation of myelin, oligodendrocytes must coordinate a plethora of neuronal activity-regulated signals that could regulate different aspects of their differentiation and myelination. For example, since peptidergic signals such as BDNF regulate oligodendrocyte proliferation, differentiation and survival; perhaps peptidergic release primarily regulates lineage progression, while synaptic vesicle fusion primarily regulates myelin formation or growth per se. Furthermore, some axonally-released neurotransmitters e.g. ATP [216], occur through volume activated anion channels [217], indicating future studies should consider non-vesicular modes of release. Recent *in vivo* studies also now show that novel CNS neurotransmitters continue to be discovered [218]; and that co-packaging and co-release of multiple neurotransmitters from the same neuron is surprisingly frequent (e.g [219,220], reviewed in [221]). Oligodendrocytes express a variety of neurotransmitter receptors that could induce different intracellular responses. For example, NMDA receptor activation in oligodendrocytes increases glucose uptake and enhances the metabolic support oligodendrocytes provide to the axon [196]. Therefore, NMDA signalling may not overtly regulate myelin structure [222], but may enable oligodendroglial metabolic support to be matched to the energy needs of the axon. Oligodendrocytes also express receptors for GABA, adenosine, or endocannabinoids, among others [77,223]. These pathways could be engaged in parallel to regulate different aspects of myelin physiology or structure.

It is also worth considering that distinct environmental stimuli, engaging different neuronal circuits, could each employ specific signalling pathways to induce their own cellular and behavioural adaptations. For example, motor skill learning, sensory enrichment, fear memory, spatial learning and working memory have all been linked to the generation of new oligodendrocytes [26,30,31,52,54–57]. In contrast, experience-dependent remodelling of existing myelin has been reported in fewer paradigms [sensory enrichment, auditory stimulation, fine motor skill learning and sensory deprivation] [24,48–51,55,56,71,224]. Studies in the zebrafish demonstrated that only certain neuronal subtypes regulate their myelination through synaptic vesicle release [70], a specificity that has also been observed in mammals in a monocular deprivation paradigm [24]. Therefore, identifying which neuronal subtypes are activated and the individual signals released during various tasks will enhance our understanding of how activity-dependent myelination supports complex behaviours.

## Final remarks and perspectives

In summary, an accumulating body of evidence points to multiple routes by which neurons could communicate activity-related signals to oligodendrocytes, enabling myelinated circuits to adapt and respond to external stimuli. We have highlighted recent *in vivo* evidence of activity-regulated vesicular fusion along axons, which we propose represents a crucial mode of neuron-oligodendrocyte signalling, and which may also represent a route used by neurons more broadly for communication with other CNS cell types. Potentially previously overlooked due to a predominant focus on synaptic forms of neuronal signalling, direct axon-to-oligodendrocyte/myelin signalling opens up an exciting new area of investigation. Furthering our understanding of axonal neurotransmission and the relative contribution of this and other ways by which neurons signal activity levels to oligodendrocytes (outlined above) will go some way towards explaining the diversity of myelinated circuits in the CNS. For example, axonal release and indeed adaptive myelination seems to be a neuron-subtype-specific property [24,69,70]. Potentially, differences in activity levels or expression of key molecular machinery for axonal release or other activity-regulated pathways could underlie this diversity.

Subsequently, adaptive changes in the pattern of myelination induced by axonal release alter the millisecond precision with which synapses connect two neurons, regulate synaptic plasticity and fine-tune network activity and ultimately brain function [69,225–227], through circuit adaptations that will be important to elucidate. It is important to note that in addition to myelin-associated adaptations to conduction, axonal release could induce oligodendrocytes to modulate synaptic properties through other mechanisms, e.g. through direct BDNF release [219], or provision of metabolic substrates to adapt to fluctuating energy demands [6,7]. For example, *de novo* myelination might increase transfer of metabolic substrates from more oligodendrocytes [71].

Ultimately, it will be crucial to understand if axonal release plays a unique role in shaping myelinated circuits during development or throughout life, and how significant it is in the human CNS, which emerging organoid models will allow us to explore [228,229]. If it is, dysregulation of axonal release could have profound significance in the pathophysiology both of human neurodevelopmental disorders and of neurodegenerative conditions in which myelination impairments have been implicated [230–232].

## Open Access

Open access for this article was enabled by the participation of The University of Edinburgh in an all-inclusive *Read & Publish* agreement with Portland Press and the Biochemical Society under a transformative agreement with JISC.

## Abbreviations

BDNF: brain-derived neurotrophic factor
CAM: cell adhesion molecule
CNS: central nervous system
ECM: extracellular matrix
GABA: γ-aminobutyric acid
Nrg1: *neuregulin 1*
OPC: oligodendrocyte precursor cell
RON: rat optic nerve
TeNT: Tetanus neurotoxin
